# Dietary patterns of Pakistani adults and their associations with sociodemographic, anthropometric and life-style factors

**DOI:** 10.1017/jns.2013.37

**Published:** 2014-01-02

**Authors:** Nilofer F. Safdar, Elizabeth Bertone-Johnson, Lorraine Cordeiro, Tazeen H. Jafar, Nancy L. Cohen

**Affiliations:** 1 School of Public Health, Dow University of Health Sciences, OJHA Campus, Suparco Road, Karachi 75270, Pakistan; 2 Department of Public Health, School of Public Health and Health Sciences, University of Massachusetts, Amherst, MA 01003-9282, USA; 3 Department of Nutrition, School of Public Health and Health Sciences, University of Massachusetts, Amherst, MA 01003-9282, USA; 4 Department of Community Health Sciences, Aga Khan University, Stadium Road, Karachi 74800, Pakistan

**Keywords:** Dietary patterns, Factor analysis, Pakistan, Life-style behaviours, COBRA, Control of Blood Pressure and Risk Attenuation, WC, waist circumference, WHR, waist:hip ratio

## Abstract

Dietary pattern analysis is an epidemiological method designed to consider the complexity of food preferences and diet patterns of populations. Few studies from South Asia have used this methodology to describe population food intake. Our objective was to identify dietary patterns and understand their associations with sociodemographic, anthropometric and life-style factors among low-income Pakistani urban adults. Dietary information was collected by a thirty-three-item FFQ and dietary patterns were derived by principal component analyses in 5491 subjects enrolled in the Control of Blood Pressure and Risk Attenuation (COBRA) study. Three dietary patterns were identified: a fat and sweet pattern characterised by fried snacks/foods, desserts, organ meats, bakery products, Pakistani bread and food purchased from outside the home; a fruit and vegetable pattern including fruits, juices, raw and cooked vegetables, lean meat and low-fat milk; and a seafood and yogurt pattern identified by prawns, fish, potatoes and yogurt. The fat and sweet pattern scores were low among older subjects, those with high BMI and waist circumference but high among females and physically active participants. The fruit and vegetable pattern was associated with younger age, high BMI, education and non-tobacco use. The seafood and yogurt pattern was associated with high BMI, increased physical activity and non-tobacco use. In conclusion, distinct dietary patterns exist for the Pakistani population that may be related to some of the population characteristics and thus may have importance in suggesting dietary and life-style interventions in the prevention of chronic diseases.

The description of a population's unique food pattern is an important step in promoting the intake of an optimal diet for disease prevention and health promotion^(^^1^^,^^2^^)^. The use of dietary pattern analysis focusing on the whole diet rather than on a single food or nutrient has numerous advantages in linking diet to health outcomes^(^^3^^,^^4^^)^. Normally, individuals consume food items composed of many nutrients and non-nutrients and dietary pattern analysis has the ability to integrate the complex and subtle interactive effects of many dietary exposures. Dietary pattern analysis may also help to resolve the problems generated by the multiple testing and high correlations that regularly occur in the analysis of individual foods and nutrients^(^^5^^,^^6^^)^. Compared with individual food intakes, dietary pattern studies may as well reflect an individual's food preferences that have been influenced by cultural, health, social, environmental, life-style and economic factors^(^^1^^)^. Most data in the area of dietary patterns and related factors have been drawn from epidemiological studies conducted in developed countries^(^^7^^–^^9^^)^. In developing countries, especially in South Asia, studies of dietary patterns are scarce^(^^10^^,^^11^^)^. There is also a dearth of information on food patterning and its relationship with various life-style characteristics in the South Asian population. Differences in genetic composition, environmental exposures, as well as the marked variations in the diets of Western and non-Western nations suggest that the results obtained from studies on the dietary patterns of Western populations cannot be extrapolated to developing countries^(^^11^^–^^14^^)^. The few studies conducted thus far on dietary patterns in Pakistan have used a relatively small, non-representative sample and were limited to specific diseases^(^^11^^,^^14^^)^. Furthermore, the knowledge gap also remains in relation to the characteristics and variations in Pakistani dietary patterns. The primary objective of the present study was to define distinct dietary patterns and to examine their relationship with sociodemographic, anthropometric and life-style characteristics among a representative population of low-income urban adults in Pakistan.

## Materials and methods

### Population and study design

The data for the present study were collected in 2004 as part of the Control of Blood Pressure and Risk Attenuation (COBRA) study (clinicaltrials.gov no. NCT00327574). This was a population-based randomised controlled trial with a primary focus on assessing the effectiveness of community-based strategies to prevent and manage high blood pressure among low-income urban families in Karachi, Pakistan. The study followed a stratified multistage random sampling design to represent the low-income Pakistani population over 5 years of age. The population was further divided into three groups: children aged 5–14 years; younger adults from 15–39 years; and older adults from 40 years and above. The sampling procedure involved randomly selecting twelve out of 4200 lower middle-income (average household income less than $70/month) geographical census-based household clusters. Each cluster included approximately 250 houses with an average household size of seven individuals. In all consenting households, one child aged 5–14 years, one adult between 15 and 39 years and all subjects aged >40 years were randomly selected for clinical assessment. Poorly mobile patients, those mentally incompetent and unable to give consent, or patients who had known advanced liver or kidney failure or pregnancy were excluded from the COBRA study sample. Further details about the COBRA study have been published elsewhere^(^^15^^)^. Because of potential dietary pattern differences across age, the analysis reported in the present paper is limited to adults aged 15 years and above (*n* 5491). Baseline demographic, socio-economic, dietary and health-related data were collected by trained interviewers, in person at participants' homes using a structured, pre-tested questionnaire that was translated into local languages.

The COBRA study was conducted according to the guidelines laid down in the Declaration of Helsinki and all procedures involving human subjects were approved by the Ethics Review Committee of the Aga Khan University, Karachi. The present analysis of the COBRA data was reviewed and approved by the Internal Review Board of the University of Massachusetts, in Amherst. Written informed consent was obtained from all subjects.

### Dietary assessment

Dietary data were collected using a thirty-three-item FFQ. This was developed by a nationally known nutrition researcher based on the most frequently used Harvard FFQ format^(^^16^^,^^17^^)^, review of the available literature and information gathered through an informal dietary survey to reflect the underlying dietary habits of the low-income population in Pakistan. Using preliminary information, a well-structured and extensive questionnaire was formulated by keeping in mind the objectives of the COBRA study. The questionnaire was pre-tested for input on content, language clarity and layout. Revisions were made accordingly for final data collection from the study cohort. For each food item, subjects were asked how frequently they had consumed the food without specifying the portion size. Participants could specify the number of times per d, per week or per month. Food intake frequencies were then standardised to number of times per d. For example, a response of 2·50 times/week was converted to 0·35 times/d.

### Life-style assessment

Information on physical activity was obtained using the International Physical Activity Questionnaire^(^^18^^)^. Participants were asked to identify the frequency and duration of walking, as well as moderate and vigorous activities during the last 7 d. Average weekly duration of each activity was computed and metabolic equivalent tasks (MET)/week were calculated using Ainsworth's compendium^(^^19^^,^^20^^)^. Survey questions assessed participants' tobacco and smoking-related habits (i.e. use of cigarettes/cigars/*biddi*, pipe/*huqqah*, *niswar* and chewing tobacco with or without *pan* leaf). Participants were classified as non-tobacco users if they had never used tobacco in any form, as past tobacco users if they had used tobacco in the past but had stopped in the last year or longer, or current tobacco users if they were currently using tobacco in any form.

### Anthropometric assessment

Anthropometric measurements were collected by trained technicians following standard procedures that have been described elsewhere^(^^15^^)^. Weight was measured to the nearest 0·1 kg (Tanita Solar Powered Digital Scale model 1631; Tanita) and height to the nearest 0·5 cm (portable stadiometer). BMI was calculated as weight in kg divided by the square of height in metres. Abdominal obesity was assessed by waist circumference (WC) and was measured with a non-stretchable measuring tape at the narrowest diameter between the costal margin and the iliac crest. Hip circumference was measured at the greatest diameter over the hip. Waist:hip ratio (WHR) was calculated as WC divided by hip circumference in cm.

The WHO population cut-offs for Asians were used to classify subjects as: underweight = BMI < 18 kg/m^2^, normal = BMI 18–22·9 kg/m^2^, overweight = BMI 23–<25 kg/m^2^ and obese = BMI ≥ 25 kg/m^2^. Males with WC ≥ 90 cm and females ≥80 cm were considered as having a suboptimal WC. WHR values ≥1·0 for men and ≥0·85 for woman were considered elevated^(^^21^^)^.

### Statistical analysis and dietary pattern derivation

The characteristics of the study population are reported as mean values and standard deviations for continuous variables (age, WC, WHR and BMI) and percentages for categorical variables (sex, marital status, education, occupation, physical activity and tobacco use). Group differences were analysed using independent-samples *t* tests for continuous variables and the χ^2^ test for categorical variables. A total of thirty-three food items of the FFQ were included in the principal component factor analyses to identify independent dietary patterns^(^^22^^,^^23^^)^. Before using factor analysis, the suitability of this method was assessed using Kaiser–Meyer–Olkin (KMO) statistics and the Bartlett test of sphericity (BTS). An eigenvalue > 1·50 criterion, scree plot and percentage variance were used to determine the final number of factors^(^^24^^,^^25^^)^. The factors were then orthogonally rotated using the Varimax procedure to improve factor interpretation. To describe the dietary pattern within each factor component, a component matrix was created, and food items with a factor loading of > 0·30 were considered most informative in describing that factor. High loadings indicated stronger associations between the corresponding food item and dietary factors. Factors were thereby interpreted as dietary patterns and named after the foods with the highest factor loadings characterising the pattern^(^^5^^,^^26^^,^^27^^)^. A respondent's factor scores on each dietary pattern were calculated by multiplying the factor loadings of that pattern with the respondent's standardised daily frequency of food consumption added up over each food item^(^^25^^)^. The factor scores represent the level of adherence to that specific pattern, with a mean of 0, a positive score indicating adherence, and a negative score indicating avoidance of that dietary pattern. We initially derived dietary patterns in the entire study population, and then performed a separate factor analysis stratified by sex to verify the consistency among subgroups. For each pattern, participants were grouped by quartiles of dietary pattern scores. Mean intakes of key food items (>0·30 factor loading) across quartiles of dietary patterns were calculated in order to understand the composition of each dietary pattern (Supplementary Table S1). Group differences among study population across dietary pattern quartiles were analysed using ANOVA for continuous variables and by the χ^2^ test for categorical variables. To find the direction of the linear relationship between dietary pattern scores and continuous variables (age, BMI, WC and WHR), we used the Pearson correlation coefficient (Supplementary Table S2). Linear regression models with 95 % CI were built with sociodemographic, anthropometric and life-style factors as independent variables (age and BMI as quantitative, and sex, marital status, education, tobacco use and physical activity as qualitative data) and factor scores of the dietary patterns were taken as continuous variables. These models were adjusted for age and sex. All analyses were performed using the Statistical Package for the Social Sciences (version 16.0; SPSS Inc.) and *P* < 0·05 was regarded as statistically significant.

### Results

Table 1 summarises the sociodemographic, anthropometric and life-style characteristics of the study participants stratified by sex. Out of 5491 subjects, 57 % were over 40 years of age, 66 % were married, and 57 % were overweight or obese. The average age of the study participants was 40·5 (sd 15·8) years and, compared with women, men were more likely to have more years of formal education, be employed, use tobacco and be more physically active at the time of survey.

**Table 1.** General characteristics of the participants of the Control of Blood Pressure and Risk Attenuation (COBRA) study at baseline, 2004 (*n* 5491) (Mean values and standard deviations, or percentages)

Characteristics	Men (*n* 2500)	Women (*n* 2991)	*P*
Age (years)			<0·01
Mean	41·2	40·1	
sd	16·1	15·6	
BMI (kg/m^2^)			<0·01
Mean	23·3	25·4	
sd	4·8	5·9	
Waist circumference (cm)			<0·01
Mean	86·4	82·8	
sd	14·0	13·7	
Waist:hip ratio			<0·01
Mean	0·92	0·83	
sd	0·1	0·1	
Education (%)			<0·01
No formal education	16·6	33·4	
Primary–middle school	36·0	28·0	
Secondary–intermediate	32·5	28·9	
Graduate and above	14·8	9·7	
Marital status (%)			<0·01
Single	27·9	19·2	
Married	68·4	64·5	
Divorced/separated/widowed	3·7	16·3	
Occupation (%)			<0·01
Employed	76·1	12·3	
BMI* (%)			<0·01
<18 kg/m^2^ (underweight)	14·4	9·9	
18–22·9 kg/m^2^ (normal weight)	35·6	26·6	
23–24·9 kg/m^2^ (overweight)	15·8	14·7	
≥ 25 kg/m^2^ (obese)	34·1	48·7	
Waist circumference (%)			<0·01
Suboptimal/risk for disease†	40·2	57·5	
Waist:hip ratio (%)			<0·01
Suboptimal/risk for disease‡	15·1	40·7	
Physical activity (%)			<0·01
< 5 MET h/week	28·7	46·4	
5–10 MET h/week	17·2	15·2	
>10 MET h/week	54·2	38·4	
Tobacco use§ (%)			<0·01
Non-tobacco user	39·8	78·4	
Current tobacco user	48·7	19·0	
Past tobacco user	11·4	2·5	

MET, metabolic equivalent task.

* Asian-specific criteria.

† Waist circumference: male ≥90 cm and female ≥80 cm (Asian-specific criteria).

‡ Waist:hip ratio: male ≥1·0 and female ≥0·85 (Asian-specific criteria).

§ Tobacco use: those who neither smoke nor used tobacco ever are non-tobacco users; current users are those who smoke or chew tobacco at the time of the survey; past tobacco users are those who have smoked or chewed tobacco before the time of the survey.

The factor analysis procedure indicated suitable dependability, with a KMO of 0·75 and the BTS being statistically significant (*P* < 0·001), thus suggesting that the correlation among the variables was sufficiently strong for factor analysis. Three major dietary patterns were extracted from the data and the factor-loading matrices are reported in Table 2. The ‘fat and sweet’ pattern was characterised by *tandoori nan* (yeast flat bread), *biryani*/*palou* (rice with some form of meat cooked in oil/*ghee* with spices), *halwa puri* (sweets rich in *ghee* and sugar), *kata-kat* (organ meats cooked in high fat), fried foods, milk desserts, sweets, bakery products, and foods from outside, such as pizza, burgers, *kata-kat*, *nihari*, *karahi*, etc. The ‘fruit and vegetable’ pattern was characterized by a high intake of fruits, vegetables, chicken, mutton and low-fat milk. The ‘seafood and yogurt’ pattern was identified by fish, prawns, potatoes and yogurt. Sex-specific factor analysis showed high consistency with each other (data not shown), and with those obtained using the entire dataset. Results of the combined factor analysis for both sexes were used in the present study.

**Table 2.** Factor-loading matrix for the three dietary patterns identified by factor analysis (*n* 5491)*

	Dietary patterns		
Food items	Fat and sweet	Fruit and vegetable	Seafood and yogurt
Eggs	0·27	0·17	0·03
*Paratha*	0·29	−0·10	−0·11
*Tandoori nan*	0·38†	0·04	0·11
*Halwa puri*	0·33†	−0·12	0·10
Whole milk	0·26	0·05	−0·12
Milk without cream	−0·10	0·40†	−0·01
Cream	0·19	0·02	−0·11
Milk dessert	0·32†	0·18	0·17
Ice cream	0·44†	0·24	0·06
*Dahi* (yogurt)	0·03	−0·36	0·51†
*Lassi* (yogurt drink)	0·29	0·29	−0·01
Salty *lassi* (yogurt drink with salt)	−0·08	0·18	0·50†
Margarine	0·23	0·28	−0·16
Butter	0·06	0·15	−0·01
Mutton	−0·04	0·37†	0·05
Beef	0·34†	−0·10	−0·24
Chicken	0·17	0·39†	0·13
Fish	0·03	0·02	0·63†
Prawns	0·04	−0·10	0·66†
Organ meats	0·35†	0·01	0·16
Food purchased from outside‡	0·54†	−0·04	0·01
Cooked vegetables	−0·25	0·30†	−0·17
Potatoes	0·16	−0·11	0·40†
Raw vegetables	0·07	0·53†	0·08
*Biryani*/*palou*	0·42†	0·10	0·15
Beans/lentils/dhal/peas	−0·07	0·19	−0·14
Fruits	0·09	0·60†	0·14
Fresh fruit juices	0·23	0·49†	−0·06
Bakery products	0·31†	0·10	0·14
*Mitai*/*halwa* (Pakistani desserts)	0·34†	0·11	−0·02
Fried snacks	0·48†	0·02	0·09
Nuts	0·35†	0·18	−0·11
Chocolate	0·35†	0·04	−0·01
Variance explained (%)	7·7	6·3	5·9

* Extraction method: principal component analysis, Varimax rotation converged in six iterations.

† Foods >0·30 loadings.

‡ Includes Pakistani foods, such as *kata-kat*, *karahi* and *nehari*, as well as burgers and pizza.

Table 3 describes the demographic and anthropometric variables across the dietary pattern quartiles. There were statistically significant differences in age (mean age 42·3 years in quartile 1 *v.* 39·6 years in quartile 4), and in anthropometric (BMI, WC and WHR) indicators across quartiles in the fat and sweet pattern. Results were similar for age and BMI in the fruit and vegetable pattern except no significance was seen for WC and WHR across the quartiles for this pattern. Similarly, significance was seen in the seafood and yogurt pattern for BMI and WHR across quartiles. The categorical information on sociodemographic and life-style variables across quartiles is presented in Table 4. Proportionately more females than males were in the highest quartile of all dietary patterns as compared with the lowest quartile within the same dietary pattern. Significantly, more physically active individuals were categorised in the highest quartile of the fat and sweet pattern as compared with the lowest quartile, 50·5 % compared with 38·6 % participants with metabolic equivalent tasks (MET) >10 h/week (*P* < 0·01; χ^2^ test). The number of participants with high school and post-graduate education was high in the fourth quartile of the fruit and vegetable pattern as compared with the first quartile of the same pattern. Tobacco use was significantly less common among those in the highest quartile of the fruit and vegetable and seafood and yogurt patterns compared with the lowest quartile of each pattern (66·8 *v.* 54·9 % in fruit and vegetables, and 62·4 *v.* 55·9 % in seafood and yogurt).

**Table 3.** Baseline characteristics of participants (*n* 5491) across the quartiles (Q) of dietary patterns (Mean values and standard deviations)

	Fat and sweet pattern					Fruit and vegetable pattern					Seafood and yogurt pattern				
Characteristics	Q1	Q2	Q3	Q4	*P* trend*	Q1	Q2	Q3	Q4	*P* trend	Q1	Q2	Q3	Q4	*P* trend
Age (years)					<0·01					<0·01					0·48
Mean	42·3	40·5	39·7	39·6		41·1	41·4	40·0	39·5		41·02	40·4	39·8	40·7	
sd	16·7	15·7	15·1	15·8		16·3	15·8	15·6	15·6		16·6	15·7	15·4	15·6	
BMI (kg/m^2^)					0·03					0·01					0·02
Mean	24·5	24·5	24·4	24·1		23·8	24·7	24·5	24·6		24·0	24·4	24·6	24·6	
sd	5·7	5·4	5·6	5·3		5·6	5·8	5·3	5·4		5·7	5·4	5·5	5·5	
Waist circumference (cm)					0·01					0·27					0·91
Mean	85·2	84·3	84·3	83·8		83·6	85·3	84·3	84·5		84·2	84·5	84·7	84·2	
sd	13·8	13·9	14·0	14·2		14·1	14·3	13·2	14·1		14·1	13·6	14·0	14·0	
Waist:hip ratio					0·04					0·25					0·05
Mean	0·88	0·87	0·87	0·87		0·87	0·88	0·87	0·87		0·88	0·87	0·87	0·87	
sd	0·09	0·09	0·09	0·09		0·09	0·09	0·09	0·08		0·09	0·09	0·09	0·09	

* ANOVA was used to test the significance of the linear trend in the data across dietary pattern quartiles.

**Table 4.** Baseline demographic and life-style characteristics of participants (*n* 5491) across the quartiles (Q) of dietary patterns (Percentages)

	Fat and sweet pattern					Fruit and vegetable pattern					Seafood and yogurt pattern				
	Q1	Q2	Q3	Q4	*P* trend*	Q1	Q2	Q3	Q4	*P* trend	Q1	Q2	Q3	Q4	*P* trend
Median score	0·26	0·58	0·93	1·51	0·03	0·35	0·74	1·18	1·84		–0·03	0·16	0·35	0·81	
Sex										0·02					0·01
Female	51·9	54·3	55·8	55·8		51·9	54·1	56·2	55·6		51·3	54·9	54·8	56·9	
Marital status					0·56					0·34					0·95
Single	22·4	22·7	22·5	25·1		22·9	21·1	24·0	24·8		23·5	22·6	23·1	23·6	
Married	64·0	66·5	68·9	65·7		65·2	67·4	65·5	66·8		64·9	67·5	67·2	65·4	
Widowed/divorced	13·6	10·8	8·7	9·2		11·9	11·4	10·5	8·4		11·6	10·0	9·7	11·0	
Formal education (%)					0·76					<0·01					0·90
Primary–middle	45·2	41·0	42·7	41·7		49·8	45·3	41·4	35·5		41·4	44·7	40·7	43·8	
Secondary–intermediate	38·9	40·8	41·2	43·3		36·5	41·2	43·0	43·2		44·4	38·3	41·7	40·4	
Graduate and above	15·9	18·2	16·0	14·9		13·6	13·6	15·7	21·3		14·5	17·0	17·6	15·8	
Tobacco use (%)†						45·1				<0·01					<0·01
Yes	40·6	37·7	37·5	40·7	0·97		40·5	37·7	33·2		44·1	39·9	35·2	37·6	
Physical activity (%)					<0·01					0·77					<0·01
<5 MET h/week	44·3	40·0	35·9	3·0		38·6	38·6	37·2	38·8		43·1	41·1	38·1	31·3	
5–10 MET h/week	17·1	15·1	15·8	16·4		14·8	16·0	18·0	15·6		17·4	14·9	15·8	16·4	
> 10 MET h/week	38·6	44·9	48·3	50·5		46·6	45·4	44·8	45·6		39·5	44·1	46·1	52·6	

MET, metabolic equivalent task.

* The χ^2^ test was used to find linear association across quartiles and *P* for trend is reported.

† Includes both past and current users.

Table 5 shows the results of multiple linear regression analysis between dietary pattern scores and quantitative and qualitative variables. These models were adjusted for age and sex. The fat and sweet pattern scores were negatively associated with an increase in age, BMI, physical inactivity and male participants. Conversely, a positive association was seen for this pattern among single and bachelor participants. For the fruit and vegetable pattern, a negative coefficient was observed for age, those who were widowed, separated or divorced, had primary education and were smokers, whereas a positive coefficient was seen with BMI. The seafood and yogurt pattern was negatively associated with males, tobacco use and physical inactivity.

**Table 5.** Sociodemographic and life-style factors associated with adherence to the major dietary patterns among Pakistani adults (*n* 5491) (β Coefficients and 95 % confidence intervals)

	Fat and sweet		Fruit and vegetable		Seafood and yogurt	
	β†	95 % CI	β	95 % CI	β	95 % CI
Age (years)	−0·02**	−0·03, 0·00	−0·02**	−0·03, 0·00	0·01	0·01, −0·01
Sex						
Female (*n* 2991)	0	Reference	0	Reference	0	Reference
Male (*n* 2500)	−0·04**	−0·07, –0·01	−0·02	−0·05, 0·01	−0·04**	−0·06, –0·02
Marital status						
Married (*n* 3638)	0	Reference	0	Reference	0	Reference
Single/bachelor (*n* 1273)	0·08**	0·02, 0·14	0·01	−0·06, 0·07	0·02	−0·02, 0·07
Divorced/widowed/separated (*n* 580)	−0·06	−0·13, 0·01	−0·09*	−0·16, −0·02	−0·03	−0·07, 0·02
Formal education						
Graduate and above (*n* 662)	0	Reference	0	Reference	0	Reference
Secondary–intermediate (*n* 1676)	0·04	−0·04, 0·12	−0·10	−0·19, 0·01	−0·01	−0·06, 0·05
Primary–middle (*n* 1737)	0·05	−0·03, 0·13	−0·21**	−0·30, −0·12	0·03	−0·03, 0·08
BMI (kg/m^2^)	−0·04**	−0·07, –0·01	0·04**	0·01, 0·07	−0·01	0·01, −0·02
Tobacco use						
No (*n* 3342)	0	Reference	0	Reference	0	Reference
Yes (*n* 2149)	−0·02	−0·08, 0·03	−0·14**	−0·19, −0·08	−0·04*	−0·08, 0·00
Physical activity (MET h/week)						
>10 (*n* 2501)	0	Reference	0	Reference	0	Reference
10 to 5 (*n* 883)	−0·05	−0·11, 0·02	0·04	−0·03, 0·11	−0·09**	−0·13, –0·04
<5 (*n* 2102)	−0·12**	−0·17, –0·08	0·02	−0·03, 0·07	−0·17**	−0·20, –0·14

MET, metabolic equivalent task.

* *P* < 0·05, ** *P* < 0·01·

† The β regression coefficient was calculated using a linear regression model, adjusted for age and sex. For age and BMI the β coefficient has been multiplied by 10 to improve the interpretability of small β values for readers.

## Discussion

To our knowledge, this is the first study to report specific dietary patterns among a low-income urban people in Pakistan and to determine their associations with sociodemographic, anthropometric and life-style characteristics of subjects. In the present cross-sectional study, three distinct dietary patterns were identified explaining approximately 20 % of the variance in food intake among adults who participated in the COBRA study. While these dietary patterns may be specific to an urban Pakistani population, they did not differ much in the context of food items and food groups from dietary patterns derived in other population groups^(^^28^^,^^29^^)^.

Dietary pattern analysis is useful in evaluating the multidimensional nature of diets. Previous researchers have reported different dietary patterns derived from FFQ by using factor analysis methodology^(^^9^^,^^10^^,^^27^^,^^30^^)^. For example, the hydrogenated and saturated fat and vegetable oil pattern identified among the West Bengali population had similar food items to the fat and sweet pattern of the present study^(^^10^^)^. Among the Iranian population, Rezazadeh *et al.*^(^^12^^)^ identified healthy and unhealthy patterns. Their healthy pattern was characterised by a high intake of vegetables, fruits, yogurt drinks, poultry, fruit juice, potatoes and coffee. Similarly, in the present study, foods such as vegetables, fruits, fruit juice and chicken were included in the fruit and vegetable pattern. The Iranian unhealthy diet pattern was identified by organ meats, sweets, snacks, refined grains, nuts and high-fat dairy products. Some of these foods were similar to the foods included in the fat and sweet pattern in the present study. Studies of Western dietary patterns have also identified similar type of patterns^(^^26^^,^^30^^,^^31^^)^. To enable comparability of Western dietary patterns with the Pakistani fat and sweet pattern, we found an increased intake of burgers, fried foods, pizza and chips in the processed diet pattern and sweets, bakery items and chocolate in the confectionery dietary pattern of British people^(^^26^^)^. Similarly, Sofianou *et al.*^(^^31^^)^ identified a high intake of meats, desserts and fried potatoes in the Western diet pattern of Mexican–American adults. Although specific food items or prepared forms of dishes may differ in populations due to cultural preferences, cooking style and cuisine, food ingredients often remain the same.

Although seafood is expensive in Pakistan, we identified a dietary pattern including fish, prawns and yogurt among the study population. This is because Karachi is a seaport city with access to a variety of fish, prawns and other seafood. Dietary preferences in coastal communities combined with low-grade and/or low-cost varieties of seafood and the use of yogurt in local cuisine may explain factor loadings of this pattern.

Population studies have shown that social factors can also influence diet patterns^(^^12^^,^^30^^,^^32^^,^^33^^)^. The present study showed that there were significant sex differences across the quartiles of dietary patterns. For women, an increasing trend across quartiles was observed in all three dietary pattern scores as compared with men in the univariate model; however, this trend remained significant in the fat and sweet and the seafood and yogurt patterns in multivariate adjusted analyses. Contrary to findings from previous empirical studies^(^^34^^,^^35^^)^, a higher number of women were following the fat and sweet pattern as compared with men in the present study. Previous studies in different populations have also suggested that patterns may vary between the sexes but often with women consuming fruits and vegetable and healthier diets^(^^36^^,^^37^^)^. Further prospective studies are needed in the Pakistani population to investigate if specific dietary patterns are truly associated with sex.

The hypothesis that dietary patterns might vary due to age and between younger and older adults has also been suggested by previous studies^(^^5^^,^^37^^)^. Similar results were observed in our dataset with significant differences in mean age across quartiles in the fat and sweet and fruit and vegetable patterns. Besides age, a relationship was also observed between qualitative variables and dietary pattern scores. There was a greater adherence to the fruit and vegetable pattern among those who had more years of education, which is similar to other studies in different populations^(^^5^^,^^12^^,^^38^^)^. Generally, an increase in education level results in a better socio-economic status (SES) and higher purchasing power. Education also enables individuals to obtain information about health and nutrition to improve health-related behaviours. Furthermore, it has also been reported that an increased SES in Pakistan is associated with a higher intake of fruits, vegetables, lamb and chicken^(^^39^^)^. Consistent with previous research^(^^11^^,^^40^^)^, the present study showed negative associations of the fruit and vegetable dietary pattern with tobacco use and physical inactivity.

Contrary to Western research, where a high intake of fruits and vegetables is associated with a lower risk of obesity^(^^41^^–^^43^^)^, we found a higher mean BMI among those who were placed in quartile 4 of the fruit and vegetable pattern as compared with those in quartile 1 of the same pattern. When similar associations were examined with dietary pattern scores instead of quartiles in the multivariate linear analysis, results were similar. It is important to examine the various reasons that could possibly relate to positive associations between vegetable and fruit intake and increased BMI in the Pakistani population. However, results similar to those of the present study have also been reported in dietary pattern studies in an East Asian population with regard to vegetable dietary patterns and obesity^(^^44^^)^. These observations could partly be explained by East Asian cooking methods^(^^11^^,^^44^^–^^46^^)^. Most East Asians prefer cooked *v.* raw vegetables, use plenty of fat during cooking and like to stir-fry their vegetables. However, due to the limitation of the present study, we do not have information on food portion sizes, individual fat and energy intake, and thus cannot postulate that comparable reasons could apply to Pakistani adults following the fruit and vegetable pattern. However, our findings are consistent with some of the previous research^(^^11^^,^^40^^)^. The INTERHEART study, which included a Pakistani population, showed that WHR increased with improved intake of vegetables and fruits^(^^45^^)^. In addition, other studies from Pakistan have also shown an increased intake of fats in daily cooking^(^^47^^–^^49^^)^. Nonetheless, the present study showed that with an increased intake of fruits and vegetables, one is more likely to refrain from unhealthy behaviours such as tobacco use and physical inactivity. These results concur with previous research^(^^11^^,^^40^^)^.

The fat and sweet pattern in the present study included typical Pakistani foods, such as *tandoori nan*, *biryani/palou*, *halwa puri*, *kata-kat*, fried foods, milk desserts, Pakistani sweets, bakery products and foods from outside, such as pizza, burgers, etc. These food items are fairly similar in food ingredients to previously described unhealthy^(^^12^^)^, Western^(^^37^^,^^43^^)^ or processed food^(^^43^^)^ eating patterns in various cross-sectional and prospective studies. Although the fat and sweet pattern in the present study was not truly a Western-type dietary pattern that typically would include more processed food, refined grains, desserts, French fries and carbonated beverages^(^^1^^,^^50^^,^^51^^)^, nonetheless, food ingredients seem similar in both patterns. Studies of Western populations have usually reported a positive associations between anthropometric measurements and fat and sweet-type dietary patterns^(^^37^^,^^52^^)^. We had postulated that higher scores in the fat and sweet pattern would be associated with overweight and abdominal adiposity in our data. The present study results were in contrast to those seen in Western populations in terms of the fat and sweet dietary pattern and body size^(^^24^^,^^53^^–^^55^^)^. However, some studies have also shown that dietary patterns categorised as fatty, sweet or energy-dense may not be associated with increased BMI^(^^56^^–^^58^^)^. One of the most important reasons for the difference could be that we did not have any energy intake data to evaluate the relationship between the fat and sweet dietary pattern and anthropometric parameters. Second, it is possible that in our population those with high fat and sweet pattern scores were also more physically active than those with low scores on the fat and sweet pattern. Third, portion size could be a factor, as average food portion sizes in Asian populations are much smaller than those found in Western populations^(^^10^^)^. Thus, future studies are needed to examine food portion sizes and calculate energy intake in studies of dietary patterns.

Clustering of life-style habits with different diet patterns has been reported previously, showing that a healthy diet leads to healthy behaviours and vice versa^(^^13^^,^^38^^)^. In the present study, we found a lower proportion of the population in both the fruit and vegetable and seafood and yogurt patterns who smoked. Similarly, in multivariate analysis, negative associations were seen between these patterns and smoking and physical inactivity. Similar results have also been shown by the INTERHEART^(^^45^^)^ study that included participants from fifty-two countries and by Yakub *et al.*^(^^11^^)^ in a Pakistani population. Their results showed that as these patterns scores increased, BMI increased as well.

The strengths of the present study include a population-based multistage random sample, a large sample size, a good response rate (88·38 %), and multiple data on sociodemographic, anthropometric and life-style characteristics that have not been previously studied in relation to dietary patterns in any other population in Pakistan. There were several limitations of the present study. First, the cross-sectional nature of the study did not allow us to establish any causality but only to test the associations between dietary patterns and various subject characteristics. Second, the modified version of the COBRA FFQ was not validated in the Pakistani population. We recognise this limitation and recommend that future studies should validate this FFQ for use in Pakistan. However, the study investigators responsible for constructing the FFQ in the COBRA study were knowledgeable about the eating patterns of the study population and used the modified Harvard FFQ that reflected ethnic-specific Pakistani foods. They further pre-tested the FFQ in a similar population to check its clarity, consistency and suitability for use in the COBRA study. Third, the serving sizes of food items were not recorded in the FFQ that may have led to over- or under-reporting, an inherent problem in studies of dietary intake^(^^59^^)^. Previous studies have suggested that portion size questions do not necessarily improve the performance of the FFQ and the source of variation in the diet may be greater in terms of differences in frequency of intake compared with differences in portion size between individuals^(^^60^^–^^62^^)^. It is difficult to quantify misreporting based on the available data, but it is likely that over-reporting by women and under-reporting by overweight men could be due to several reasons that we were unable to capture due to methodological limitations of secondary analysis. However, we cannot eliminate the influence of Asian culture and tradition playing some role in collecting dietary data from the COBRA study population. Lastly, factor analysis is a data-driven method to derive dietary patterns and the nature of the derived patterns are specific to the population under study^(^^1^^)^. This may well mean that in a different population, or even in the same population at a different time, we might have observed different patterns, which can limit the interpretability and generalisability of these dietary patterns. However, because our sample size was large and representative of the population under study, we are confident that these patterns may truly reflect low-income urban Pakistani adults.

In conclusion, few studies in South Asia have used dietary pattern analysis to understand diet and its relationship with various characteristics of the population. In the present study, three distinct dietary patterns were identified for low-income urban adults of Pakistan. These dietary patterns were associated with sex, age, education and some of the anthropometric and life-style variables. Thus, our findings support the assumption that dietary patterns differ according to populations and they may be interrelated with sociodemographic and life-style factors. In future studies, this information may hold relevance in understanding the diet–disease relationship among Pakistanis who are susceptible to chronic diseases, undernutrition, overnutrition, population growth, urbanisation, food insecurity and poor healthcare infrastructure^(^^63^^–^^65^^)^.

## Supplementary Material

Supplementary Material Supplementary information supplied by authors.

## Supplementary material

To view supplementary material for this article, please visit http://dx.doi.org/10.1017/jns.2013.37
