# Supplementary material for: Dietary patterns of Pakistani adults and their associations with sociodemographic, anthropometric and life-style factors
Source: J Nutr Sci. 2014 Jan 2;2:e42. doi: 10.1017/jns.2013.37 (PMC4153335; doi:10.1017/jns.2013.37)
Supplement: Supplementary Material — Supplementary information supplied by authors. [file S2048679013000372sup001.doc]

**Supplementary Table S1**. Daily mean Frequency intake of some key food items across the

quartiles of dietary patterns

| Food Items | Q1 | Q2 | Q3 | Q4 |
| --- | --- | --- | --- | --- |
|  | Fat and Sweet Pattern | | | |
| Tandoori Nan | 0.12 | 0.14 | 0.18 | 0.25 |
| Halwa Puri | 0.03 | 0.04 | 0.05 | 0.07 |
| Milk Dessert | 0.05 | 0.06 | 0.08 | 0.13 |
| Ice cream | 0.05 | 0.06 | 0.07 | 0.11 |
| Beef | 0.22 | 0.23 | 0.29 | 0.36 |
| Organ Meats | 0.05 | 0.06 | 0.07 | 0.10 |
| Eating Food From Outside | 0.04 | 0.05 | 0.06 | 0.09 |
| Biryani/ Palow | 0.11 | 0.13 | 0.15 | 0.18 |
| Bakery Products | 0.11 | 0.17 | 0.23 | 0.29 |
| Mitai/ Halwa (Pakistani Desserts) | 0.03 | 0.04 | 0.05 | 0.08 |
| Fried Snacks | 0.09 | 0.10 | 0.13 | 0.22 |
| Nuts | 0.06 | 0.07 | 0.11 | 0.18 |
| Chocolate | 0.10 | 0.11 | 0.12 | 0.20 |
|  | Vegetable and Fruit Patten | | | |
| Milk Without Cream/Malai | 0.10 | 0.17 | 0.23 | 0.33 |
| Mutton | 0.04 | 0.05 | 0.06 | 0.10 |
| Chicken | 0.17 | 0.19 | 0.21 | 0.30 |
| Cooked Vegetables | 0.30 | 0.33 | 0.35 | 0.38 |
| Raw Vegetables | 0.23 | 0.34 | 0.45 | 0.61 |
| Fruits | 0.23 | 0.32 | 0.48 | 0.66 |
| Fresh Fruit Juices | 0.05 | 0.07 | 0.12 | 0.23 |
|  | Seafood and Yogurt Pattern | | | |
| Fish | 0.04 | 0.05 | 0.07 | 0.13 |
| Prawn | 0.01 | 0.02 | 0.03 | 0.04 |
| Potatoes | 0.23 | 0.34 | 0.40 | 0.53 |
| Yogurt | 0.13 | 0.16 | 0.23 | 0.50 |

**Supplementary Table S2**. Correlation coefficients (*r*)^*^ between dietary pattern scores and covariates in COBRA study cohort at baseline .

| Covariates | Fat & Sweet Pattern Scores | | Fruits & Vegetable Pattern Scores | | Seafood & Yogurt Pattern Scores | |
| --- | --- | --- | --- | --- | --- | --- |
|  | *r* | *p* | *r* | *p* | *r* | *p* |
| Age in Years | -0.033 | 0.015 | -0.009 | 0.492 | 0.030 | 0.027 |
| Body mass index (kg/m^2^) | -0.042 | 0.002 | 0.026 | 0.058 | 0.033 | 0.013 |
| Waist circumference in cm | -0.024 | 0.074 | 0.025 | 0.063 | 0.016 | 0.231 |
| Waist-to-hip ratio | 0.002 | 0.899 | 0.012 | 0.360 | -0.003 | 0.799 |

*All correlations were adjusted for gender, educational status, tobacco use and physical activity.
